# Supplementary material for: Clusters of deep intronic RbFox motifs embedded in large assembly of splicing regulators sequences regulate alternative splicing
Source: bioRxiv. 2025 Mar 18:2024.08.19.608686. Preprint. [Version 2] doi: 10.1101/2024.08.19.608686 (PMC11956907; doi:10.1101/2024.08.19.608686)
Supplement: Supplement 1 — Supplemental Figure 1. Location, statistics, and fold change enrichment of the seven eCLIP peaks found across the minigene sequence shown in Figure 2. Supplementary Figure 2. Analysis of Rbfox1-eCLIP peaks in HEK293 cells with induced Rbfox1 expression (+Dox) compared to uninduced cells (No Dox). (A) Top five enriched motifs identified in CLIP-seq peaks by the HOMER motif analysis. (B) Pie chart depicting the relative frequency of eCLIP peaks that map to each specific gene region (with a peak Log2 fold enrichment ≥ 3 and p-value ≤ 0.001). (C, D) Peak Metagene Plot, depicting the average number of peaks mapped to the specific genomic regions indicated in B. The number of peaks was calculated for each gene region followed by normalization with the length of the regions. The average number of peaks was then calculated for a set number of positions along the regions. Supplementary Figure 3. Strategy to delete (T)GCATG-clusters from the TrkB-BAC minigene. (A) Schematic representation of the TrkB-BAC minigene showing the position of the PCR-primers designed to detect the deletion of (T)GCATG-cluster 1 and (T)GCATG-cluster 2 (indicated by red X) analyzed in (C). (B) magnification of Cluster 1 and 2 areas indicating the location and sequence of the primers used for the analysis. (C) PCR analysis of genomic DNA from HEK293 cells used as negative control, cell lines expressing the ‘wild-type’ TrkB-BAC minigene (77–5 and 77–6 cells), cell lines expressing the TrkB-BAC minigene with cluster 1 deletion (1–1 and 1–2 cells), cell lines expressing the TrkB-BAC minigene with cluster 2 deletion (27–51 and 27–53 cells) and cell lines expressing the TrkB-BAC minigene with both cluster 1 and 2 deletion (32–1 and 32–2 cells). The PCR detecting cluster 1 deletion shows an amplicon of 891 bp (wild-type minigene sequence) and an amplicon of 279 bp (deletion of cluster 1). The PCR detecting cluster 2 deletion shows an amplicon of 746 bp (wild-type minigene sequence) and an amplicon of 322 bp [file media-1.pdf]

Suppl. Fig. 1

Chromosome	Start	End	-log10(P-value)	Log2 Fold Change	Strand	Gene	Ensembl ID	Feature
chrTrkB_Ntrk2_minigene	31,100	31,115	400	3.14437648711126	+	Ntrk2	ENSMUSG00000055254	Distal intron
chrTrkB_Ntrk2_minigene	45,552	45,584	400	3.20890405198609	+	Ntrk2	ENSMUSG00000055254	Distal intron
chrTrkB_Ntrk2_minigene	76,182	76,233	38.9618171340093	3.77851130406743	+	Ntrk2	ENSMUSG00000055254	Distal intron
chrTrkB_Ntrk2_minigene	122,670	122,695	12.4330678000343	3.89923278816692	+	Ntrk2	ENSMUSG00000055254	Distal intron
chrTrkB_Ntrk2_minigene	142,283	142,334	21.888569448234	4.13082488341521	+	Ntrk2	ENSMUSG00000055254	Distal intron
chrTrkB_Ntrk2_minigene	142,334	142,378	15.5991167844516	3.89343880102932	+	Ntrk2	ENSMUSG00000055254	Distal intron
chrTrkB_Ntrk2_minigene	146,090	146,137	23.8935711052066	3.69845068266664	+	Ntrk2	ENSMUSG00000055254	Distal intron

Suppl. Fig. 2

A

Rank	Motif	P-value	log P-pvalue	% of Targets	% of Background	STD(Bg STD)
1	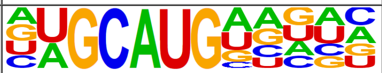	1e-2765	-6.368e+03	32.51%	7.72%	30.4bp (34.4bp)
2	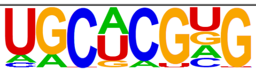	1e-155	-3.570e+02	1.41%	0.22%	25.4bp (15.7bp)
3	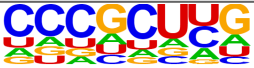	1e-146	-3.372e+02	6.32%	3.10%	35.7bp (30.9bp)
4	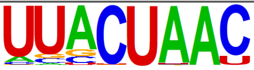	1e-132	-3.048e+02	1.41%	0.27%	32.9bp (24.5bp)
5	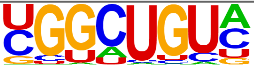	1e-127	-2.945e+02	15.36%	10.40%	33.1bp (37.8bp)

B

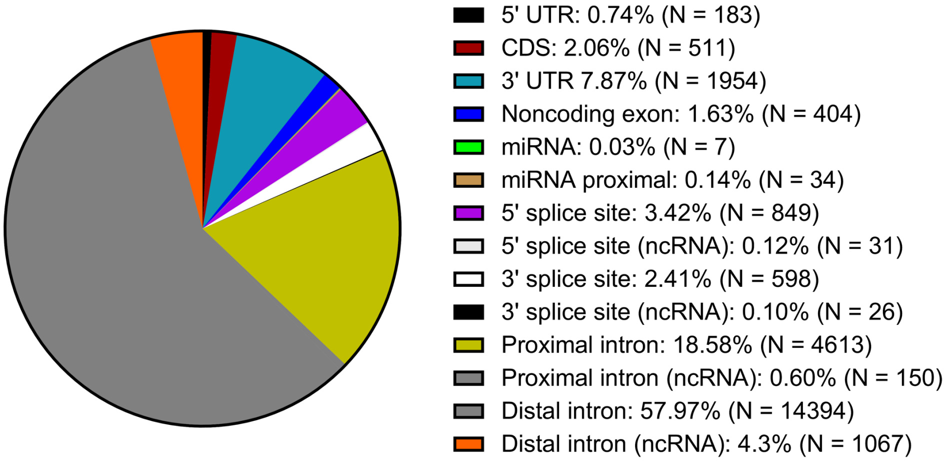

C

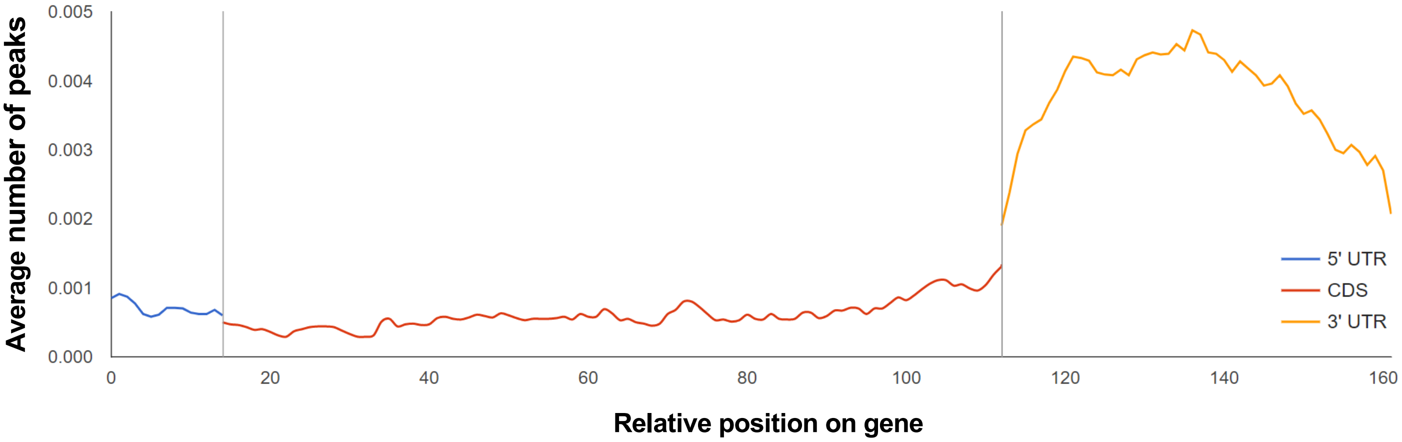

D

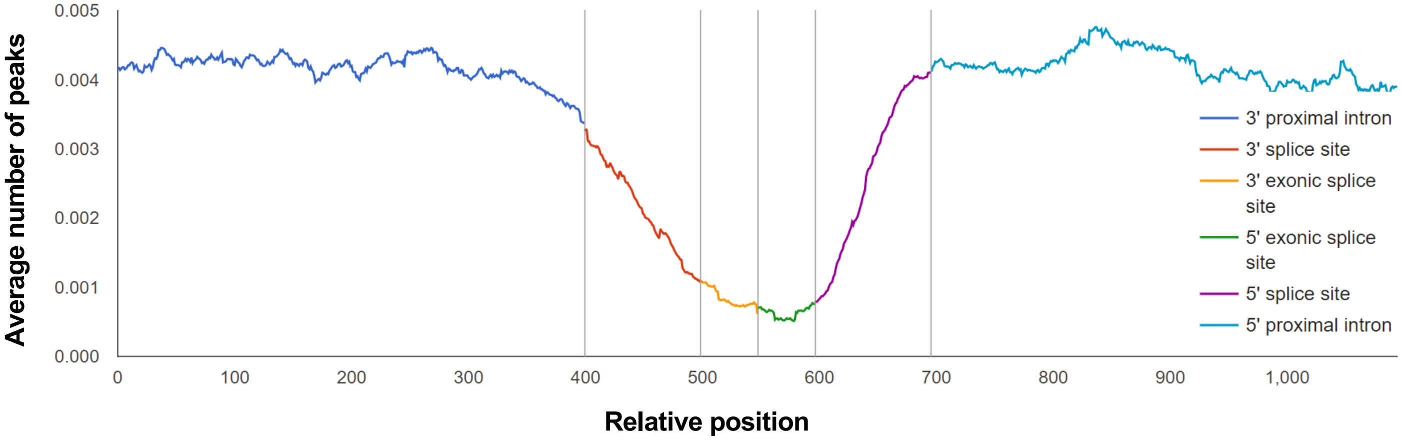

Suppl. Fig. 3

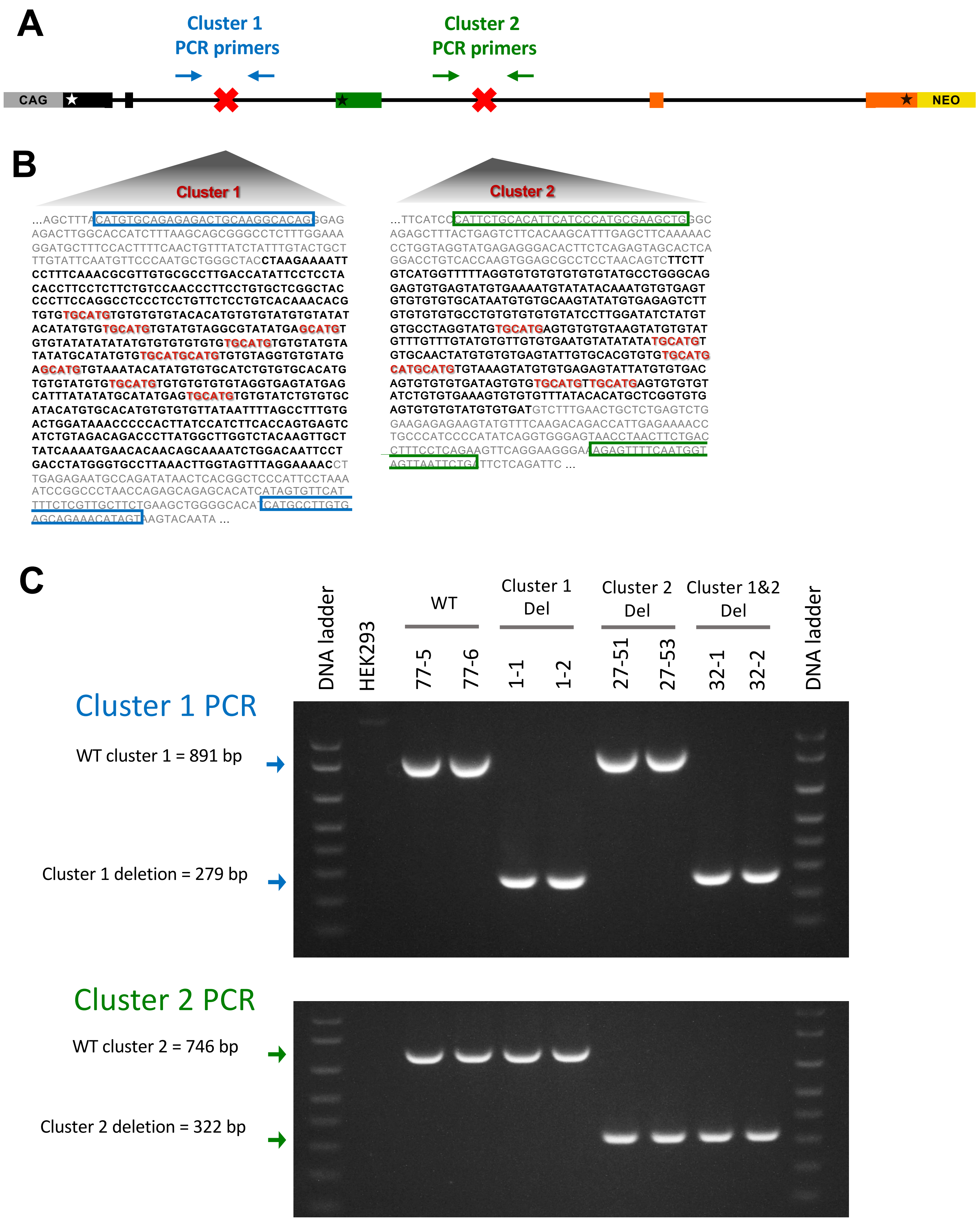

A
Cluster 1
PCR primers
Cluster 2
PCR primers
CAG
NEO
B
Cluster 1
...AGCTTTACATGTGCAGAGAGACTGCAAGGCACAGGGAG
AGACTTGGCACCATCTTTAAGCAGCGGGCCTCTTTGGAAA
GGATGCTTTCCACTTTTCAACTGTTTATCTATTTGTACTGCT
TTGTATTCAATGTTCCCAATGCTGGGCTACCTAAGAAAATT
CCTTTCAAACGCGTTGTGCGCCTTGACCATATTCCTCCTA
CACCTTCCTCTTCTGTCCAACCCTTCCTGTGCTCGGCTAC
CCCTTCCAGGCCTCCCTCCTGTTCTCCTGTCACAAACACG
TGTGTGCATGTGTGTGTGTACACATGTGTGTATGTGTATAT
ACATATGTGTGCATGTGTATGTAGGCGTATATGAGCATGT
GTGTATATATATATGTGTGTGTGTGTGCATGTGTGTATGTA
TATATGCATATGTGTGCATGCATGTGTGTAGGTGTGTATG
AGCATGTGTAAATACATATGTGTGCATCTGTGTGCACATG
TGTGTATGTGTGCATGTGTGTGTGTGTAGGTGAGTATGAG
CATTTATATATATGCATATGAGTGCATGTGTGTATCTGTGTGC
ATACATGTGCACATGTGTGTGTTATAATTTTAGCCTTTGTG
ACTGGATAAACCCCCACTTATCCATCTTCACCAGTGAGTC
ATCTGTAGACAGACCCTTATGGCTTGGTCTACAAGTTGCT
TATCAAAATGAACACAACAGCAAAATCTGGACAATTCCT
GACCTATGGGTGCCTTAAACTTGGTAGTTTAGGAAAACCT
TGAGAGAATGCCAGATATAACTCACGGCTCCCATTCCTAAA
ATCCGGCCCTAACCAGAGCAGAGCACATCATAGTGTTCAT
TTTCTCGTTGCTTCTGAAGCTGGGGCACATCATGCCTTGTG
AGCAGAAACATAGTAAGTACAATA ...
Cluster 2
...TTCATCCCATTCTGCACATTCATCCCATGCGAAGCTGGGC
AGAGCTTTACTGAGTCTTCACAAGCATTTGAGCTTCAAAAAC
CCTGGTAGGTATGAGAGGGACACTTCTCAGAGTAGCACTCA
GGACCTGTCACCAAGTGGAGCGCCTCCTAACAGTCTTCTT
GTCATGGTTTTTAGGTGTGTGTGTGTGTATGCCTGGGCAG
GAGTGTGAGTATGTGAAAATGTATATACAAATGTGTGAGT
GTGTGTGTGCATAATGTGTGCAAGTATATGTGAGAGTCTT
GTGTGTGTGCCTGTGTGTGTGTATCCTTGGATATCTATGT
GTGCCTAGGTATGTGCATGAGTGTGTGTAAGTATGTGTAT
GTTTGTTTGTATGTGTTGTGTGAATGTATATATATGCATGT
GTGCAACTATGTGTGTGAGTATTGTGCACGTGTGTGCATG
CATGCATGTGTAAAGTATGTGTGAGAGTATTATGTGTGAC
AGTGTGTGTGATAGTGTGTGCATGTTGCATGAGTGTGTGT
ATCTGTGTGAAAGTGTGTGTTTATACACATGCTCGGTGTG
AGTGTGTGTATGTGTGATGTCTTTGAACTGCTCTGAGTCTG
GAAGAGAGAAGTATGTTTCAAGACAGACCATTGAGAAAACC
TGCCCATCCCCATATCAGGTGGGAGTAACCTAACTTCTGAC
CTTTCCTCAGAAGTTCAGGAAGGGAAAGAGTTTTCAATGGT
AGTTAATTCTGATTCTCAGATTC ...
C
DNA ladder
HEK293
WT
77-5
77-6
Cluster 1 Del
1-1
1-2
Cluster 2 Del
27-51
27-53
Cluster 1&2 Del
32-1
32-2
DNA ladder
Cluster 1 PCR
WT cluster 1 = 891 bp
Cluster 1 deletion = 279 bp
Cluster 2 PCR
WT cluster 2 = 746 bp
Cluster 2 deletion = 322 bp

Suppl. Fig. 4

A

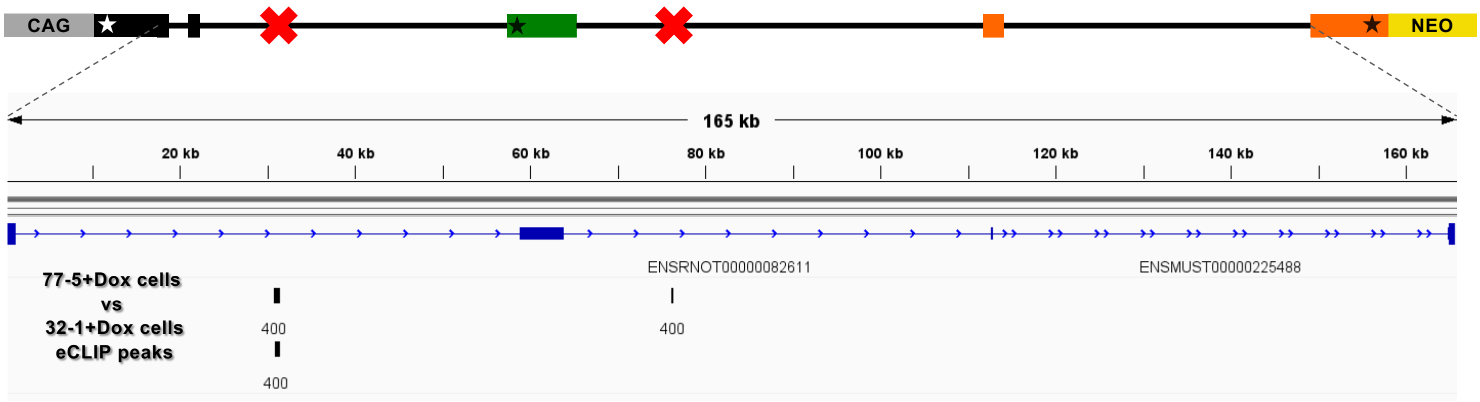

B

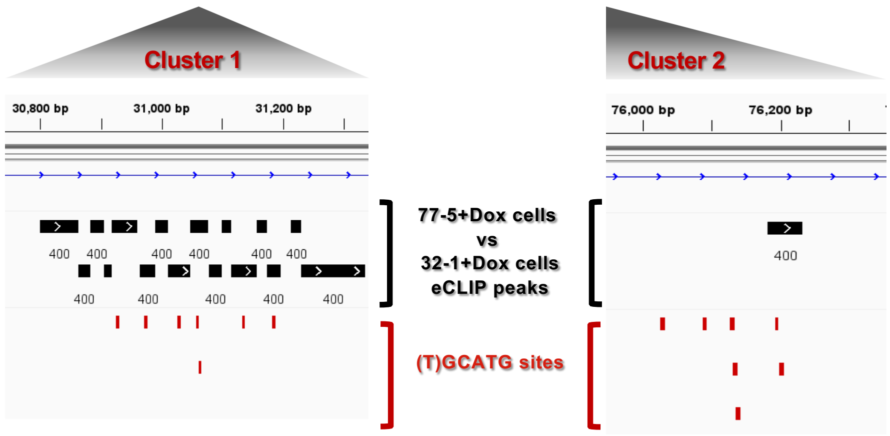

C

Chromosome	Start	End	-log10(P-value)	Log2 Fold Change	Strand	Gene	Ensembl ID	Feature
chrTrkB_Ntrk2_minigene	30,801	30,863	400	7.2027601433326	+	Ntrk2	ENSMUSG00000055254	Distal intron
chrTrkB_Ntrk2_minigene	30,863	30,882	400	7.83245626508985	+	Ntrk2	ENSMUSG00000055254	Distal intron
chrTrkB_Ntrk2_minigene	30,882	30,906	400	8.29713507889578	+	Ntrk2	ENSMUSG00000055254	Distal intron
chrTrkB_Ntrk2_minigene	30,906	30,918	400	7.94503411560365	+	Ntrk2	ENSMUSG00000055254	Distal intron
chrTrkB_Ntrk2_minigene	30,918	30,961	400	5.09097712399694	+	Ntrk2	ENSMUSG00000055254	Distal intron
chrTrkB_Ntrk2_minigene	30,964	30,990	400	5.11980751567679	+	Ntrk2	ENSMUSG00000055254	Distal intron
chrTrkB_Ntrk2_minigene	30,990	31,011	400	4.72464717782695	+	Ntrk2	ENSMUSG00000055254	Distal intron
chrTrkB_Ntrk2_minigene	31,011	31,047	400	3.69840522735328	+	Ntrk2	ENSMUSG00000055254	Distal intron
chrTrkB_Ntrk2_minigene	31,047	31,076	400	4.83377754237091	+	Ntrk2	ENSMUSG00000055254	Distal intron
chrTrkB_Ntrk2_minigene	31,078	31,100	400	6.95832944582644	+	Ntrk2	ENSMUSG00000055254	Distal intron
chrTrkB_Ntrk2_minigene	31,100	31,115	400	5.96082696528812	+	Ntrk2	ENSMUSG00000055254	Distal intron
chrTrkB_Ntrk2_minigene	31,115	31,156	400	4.41839823542178	+	Ntrk2	ENSMUSG00000055254	Distal intron
chrTrkB_Ntrk2_minigene	31,156	31,174	400	7.31839724537609	+	Ntrk2	ENSMUSG00000055254	Distal intron
chrTrkB_Ntrk2_minigene	31,174	31,197	400	6.44863628125885	+	Ntrk2	ENSMUSG00000055254	Distal intron
chrTrkB_Ntrk2_minigene	31,214	31,231	400	6.04066610949988	+	Ntrk2	ENSMUSG00000055254	Distal intron
chrTrkB_Ntrk2_minigene	31,231	31,337	400	8.14300321860379	+	Ntrk2	ENSMUSG00000055254	Distal intron
chrTrkB_Ntrk2_minigene	76,182	76,233	400	5.08149532441559	+	Ntrk2	ENSMUSG00000055254	Distal intron

Suppl. Fig. 5

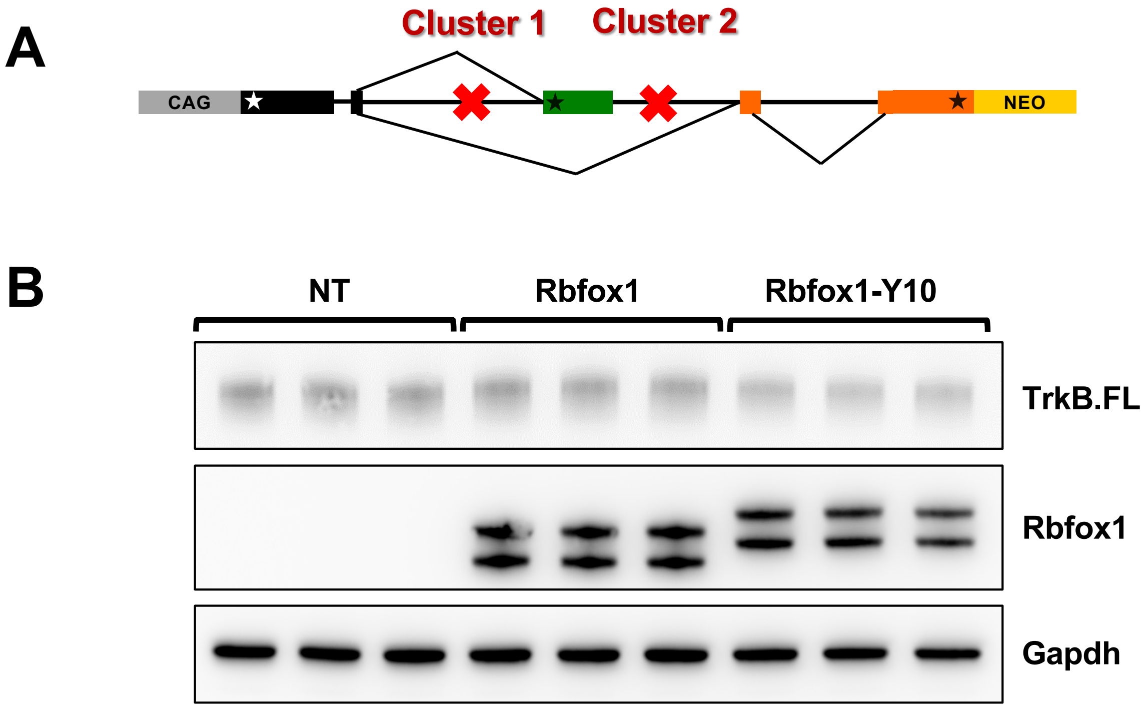
A
Cluster 1
Cluster 2
CAG
NEO
B
NT
Rbfox1
Rbfox1-Y10
TrkB.FL
Rbfox1
Gapdh
